# “The Last Thing You Have to Worry about”: A Thematic Analysis of Employment Challenges Faced by Cancer Survivors

**DOI:** 10.3390/ijerph191811214

**Published:** 2022-09-07

**Authors:** Kathleen Doyle Lyons, Rachel C. Forcino, Sivan Rotenberg, Jenna E. Schiffelbein, Kali J. Morrissette, Cassandra M. Godzik, Jonathan D. Lichtenstein

**Affiliations:** 1 Department of Occupational Therapy, MGH Institute of Health Professions, Boston, MA 02129, USA; 2 The Dartmouth Institute for Health Policy and Clinical Practice, Geisel School of Medicine, Dartmouth College, Lebanon, NH 03756, USA; 3 Department of Psychiatry, Geisel School of Medicine, Dartmouth College, Lebanon, NH 03756, USA; 4 Norris Cotton Cancer Center, Geisel School of Medicine, Dartmouth College, Lebanon, NH 03756, USA

**Keywords:** employment, cancer survivor, workplace

## Abstract

The evidence base for interventions that support the employment goals of cancer survivors is growing but inconclusive. As the first step in initiating a community-engaged program of research aimed at developing and testing interventions to support the employment goals of cancer survivors, 23 cancer survivors, 17 healthcare providers, and 5 employers participated in individual interviews to elicit perceptions regarding local challenges and resources related to work maintenance and optimization within the context of cancer treatment. Interviews were recorded and transcribed verbatim. A thematic analysis was conducted to identify cross-cutting experiences that were voiced by all three types of participants. Three themes were found in the data: (1) the onus for identifying and articulating work-related issues is upon the cancer survivor; (2) the main support offered to cancer survivors involved time away from work and flexibility with scheduling work and treatment activities; and (3) participants voiced a lack of information regarding one or more aspects related to supporting employment goals of cancer survivors. Supportive resources designed for cancer survivors, employers, and/or healthcare providers are needed to help cancer survivors optimize their employment situations.

## 1. Introduction

Forty-one percent of cancer survivors reported moderate to serious concerns about their ability to maintain paid employment in a 2020 study in the United States [1]. The ability to maintain employment can have medical implications because, within the United States, many cancer survivors obtain healthcare insurance via their workplace [2]. Similarly, the ability to work is one factor that may affect the degree to which a person experiences financial toxicity, defined as the negative impact on well-being caused by out-of-pocket costs of cancer treatment [3]. Financial toxicity can negatively affect treatment adherence [4]. As such, the National Academy of Medicine asserts that healthcare providers should collaborate with patients and employers to minimize any negative effects of cancer and its treatment on the employment outcomes of cancer survivors [5].

Employment challenges can be caused by varying and intersecting issues, e.g., fatigue, anxiety, and job demands [6]. The evidence base for interventions that support the employment goals of cancer survivors is sparse and inconclusive [7,8]. Systematic reviews of this population indicate that multi-component interventions that address physical, psychosocial, and environmental barriers to work maintenance show the most promise, but studies are often underpowered and inconclusive in their outcomes [9]. Theoretically, multi-component interventions would ideally reflect the strengths, resources, and concerns of both cancer survivors and employers in order to be maximally effective, as both have to agree upon job responsibilities and accommodations [10].

A recent research agenda to improve work outcomes emphasized the importance of soliciting and reflecting stakeholder needs and priorities when developing interventions [11]. In order to build the evidence base, we need to elicit and integrate the perspectives of three key stakeholders: cancer survivors, their employers, and healthcare providers. All three stakeholders have the potential to take action to optimize employment outcomes, yet they likely vary in their knowledge, resources, and priorities. As stated above, cancer survivors and employers need to identify mutually acceptable accommodations and work schedules. Healthcare providers can fulfil their aforementioned charge to minimize the effect of cancer by helping survivors and employers understand the variation seen in recovering from or living with cancer and the ways in which symptoms may be managed, e.g., by medication or activity and environmental adaptation.

While some studies have described the perspectives of one [10,12,13,14,15,16] of these stakeholders, reports that integrate the perspectives of all three stakeholders are rare [17,18]. Integrating these perspectives and understanding where they overlap and diverge is important because employment challenges faced by cancer survivors are often multi-faceted, stemming from and influenced by physical, psychosocial, existential, economic, and cultural factors. We sought to fill this gap with a descriptive study, collecting qualitative data from individual interviews with cancer survivors, employers, and healthcare providers. The overarching research questions were: what are the challenges faced by cancer survivors and local employers related to work maintenance and optimization, and what resources and strategies could ameliorate them? This paper presents a thematic analysis of the challenges involved in working during and after cancer treatment and the types of resources and supports that are available to or desired by participants.

## 2. Methods

### 2.1. Design

This study used a qualitative descriptive design in which we conducted individual interviews with a convenience sample of cancer survivors (target *n* = 15), healthcare providers (target *n* = 15), and employers (target *n* = 15). The study was conducted in accordance with the Declaration of Helsinki and approved by the Institutional Review Boards of Dartmouth-Hitchcock Health (STUDY02000975) and Massachusetts General Hospital (2021P002762) on 30 March 2021 and 29 October 2021, respectively. Participants provided their written informed consent prior to initiating any study activities.

### 2.2. Participants

The inclusion criteria for each type of stakeholder were as follows: (1) Cancer survivor: Any person over the age of 18 who has been diagnosed with cancer and is employed but reports needing to work fewer hours or experiencing reduced productivity at work due to their health. (2) Healthcare provider: Any person who actively provides clinical or supportive care to oncology patients. (3) Employer: Any business owner, human resources professional, or manager who oversees employees. The only exclusion criterion was living or working outside of New Hampshire or Vermont. This criterion was established because the study was conducted to inform the development of employment support for people within the catchment area of our cancer center.

Cancer survivor and employer participants were recruited through a variety of channels, including social media posts and advertisements, direct email invitations, flyers and messaging distributed through e-newsletters, and through outreach via organizations such as chambers of commerce and cancer control organizations. Healthcare provider participants were recruited through direct email invitations from the research team members who worked at the same cancer centre. At the end of each interview, the interviewer asked the participant if they would give her contact information to anyone else (e.g., their employer or another healthcare provider) who may be willing to participate in the study.

### 2.3. Data Collection

Interviews were conducted individually either in person at an academic medical center located in the Northeast region of the United States (*n* = 1) or via Zoom (*n* = 44). The interviewer was a member of the research team (K.M. or C.G.) who utilised a semi-structured interview guide customised for each type of participant (cancer survivor, healthcare provider, or employer). The guides asked each type of participant to describe (a) the challenges they face or witness when cancer survivors work during treatment or return to work after taking time off; (b) the types of conversations they have with each other about work; (c) the resources that are currently available to cancer survivors and employers; and (d) any programs, resources, or information they feel would be helpful. The interviews were recorded and professionally transcribed verbatim. A team member proofread each transcript while listening to the recording, removing names of healthcare providers or employers to maximise anonymity. The transcripts were then uploaded into NVivo software (version R1.6).

### 2.4. Analysis

This thematic analysis was guided by the approach articulated by Rubin and Rubin [19]. This involves (a) coding the transcript text with descriptive labels that capture similar ideas; (b) comparing the text within and across codes to look for similarity, variation, and nuance; and (c) searching for overarching themes that explain or describe the phenomenon of interest. In a separate manuscript, we prepared a content analysis that collates and categorizes the answers to the questions posed during the interview. In contrast, this thematic analysis revealed common experiences or opinions that we heard repeatedly voiced by all three types of respondents, often in response to different questions on the interview guide.

This thematic analysis began when one investigator (K.D.L) read all transcripts and coded the data with descriptive labels, creating an operational definition of each code. Some codes represented a priori topics of interest, such as “support from providers” or “disclosure conversations”. Other codes were derived directly from the text, such as “change in priorities” or “recovery takes time”. During the initial coding, she wrote memos detailing her evolving impressions of the data, noting themes that echoed throughout the transcripts, i.e., experiences or perspectives that were repeatedly voiced by all three types of participants. We discussed the emerging themes during research team meetings. One team member (K.D.L.) then repeatedly read the relevant coded text and wrote out a description of each theme, selecting quotations to support and illuminate the evolving interpretations. All of the co-authors then read the quotations used to explicate the theme, assessed the degree to which all three types of participants had experiences and opinions reflected in the themes, explored the range of responses, and selected the best quotations to flesh out the themes.

## 3. Results

### 3.1. Participant Characteristics

A total of 45 participants (23 cancer survivors, 17 healthcare providers, and 5 employers) responded to advertisements or personal requests and enrolled in the study, completing an individual interview. We overenrolled cancer survivors and healthcare providers due to the enthusiastic response to advertisement and snowball sampling. However, we struggled to recruit employers. When directly asked, many told us they were having a hard time recollecting any instances where their employees were undergoing treatment. Notably, three of the cancer survivors reported that they were also supervisors at work; we, therefore, incorporated questions from the employer interview guide into their interviews, allowing us to have a total of eight employer’s perspectives. Characteristics of the sample are presented in Table 1.

We identified three themes in the data generated by cancer survivors, healthcare providers, and employers: (1) The onus for identifying and articulating work-related issues is upon the cancer survivor; (2) The main support offered to cancer survivors involved time away from work and flexibility with scheduling work and treatment activities; and (3) Participants voiced a lack of information regarding one or more aspects related to supporting employment goals of cancer survivors. The themes are described below. Quotations from cancer survivors, healthcare providers, and employers are noted with “CS”, “HP”, and “E”, respectively, followed by the participant study identification number.

### 3.2. Theme 1: “All on Me”

The three types of participants generally said that the cancer survivor needs to be able to voice employment concerns and ask for help because while resources may be available, they are not always publicized and may not be offered if the cancer survivor does not initiate the conversation. A cancer survivor used the words *“all on me”* (CS, 101) when describing how neither her healthcare team nor her employer offered support or guidance regarding how she could return to work while experiencing lingering side effects from treatment. This sentiment was voiced by many cancer survivors, as articulated by one who said, “*… you have to still be the one to send up the flares so that people pay attention and can try to help you*”. (CS, 110).

There was evidence of this theme in employers’ accounts where they relied upon the cancer survivor to disclose their challenges so that they could provide support. “*I’d recommend that they, you know, that they take it as discreet as they would want to be, but… I’d recommend that they do disclose because there’s a lot we can do to help*”. (E, 302). Privacy concerns or discomfort discussing details of cancer can contribute to the onus placed on the cancer survivor to initiate and manage disclosure. “*Um, some (employees) will give you, um, the bare necessity of information and some will give you everything. Um, and I would prefer like the middle of the road. Um, because you can’t really help unless you know, really what someone’s facing. Um, or what their actual situation is. So, that makes it a little harder*”. (E, 306).

Healthcare providers also indicated that they usually relied upon cancer survivors to initiate conversations about employment concerns, not because of disinterest but because oncology care is primarily focused on the treatment of disease and the management of toxicity. One survivor stated, “*Yeah, I am sure if I brought it to their (oncology clinicians) attention they would have (addressed an employment issue), but no one even mentioned like, ‘here are some things that may help you return (to work)’—my doctors were just purely like— medicine focused, like your drain’s out, your scar’s healed, your tongue isn’t all the way healed but it’s in your mouth… you can walk and lift… you’re good to go*”. (CS, 101). A healthcare provider stated, “*So, I think financial and work-related things… for years, it (work and financial issues) was never something we (oncology clinicians) thought about, we just thought, “Oh, we cured you of your cancer. Good luck. Like, That’s great”. And we never really paid attention to the fact of what else was happening…*” (HP, 202). Even when employment issues were discussed by healthcare providers, it remained the responsibility of the cancer survivor to take action and utilize resources. “*… what we (social workers) do is like, try to get the lay of the land with what people need and then connect them to whatever it is. We’re not actually doing, we’re just bridging it. So if I had to, like, type up their resume with them… that would be, like, wow, we need an agency, you know, something for that. But if it’s just call this number, here’s what I found… And it’s just about, like, being a search engine for them…*” (HP, 209).

In addition to needing to initiate the conversation about employment needs and challenges, cancer survivors are often expected to know what they want and need to work successfully. “*I think we ask the patient whether they think they can do their job, and whatever they want, if they wanna go out on leave, we sign the paperwork. If they wanna take disability, we sign the paperwork. Like, we sort of leave it up to them, but it sort of means they’re making that decision on their own without somebody really helping them determine if they can do that work or not*”. (HP, 202). When recounting a story of how she supported a recent patient communicate with her employer, another provider stated, “*…I felt like my role was kind of, you know, just simply (ask)—when you’re at work, what are the things we can do? It was like a 10-min conversation and then typing up a letter. Um, so I really do think it was more her*”. (HP, 204).

### 3.3. Theme 2: Time Is the Main Ask and Offer

When cancer survivors, employers, and healthcare providers discussed how they dealt with employment challenges, the majority of the talk was about taking time off from work. This second theme resonated throughout all of the interviews, in that reducing hours seemed to be the most frequent request from cancer survivors and the easiest thing for an employer to offer their employee during cancer treatment. Employers and survivors talked about co-workers donating sick time or using advance sick leave policies to allow people time away from work. *“Take what you need”* (CS and E, 118) in terms of time off was a common sentiment from employers. “ *(My boss said) I will hold your job. You have nothing to worry about. You need to go out and get healthy. And, and don’t worry about your job. That’s the last thing you need to worry about*”. (CS, 116). This time away from work could benefit both the employee and the workplace. “*One of the first things anybody hired does at the company is sit with the president and talk about how important family is and how…there’s a clear structure in place to honor the fact that if you are not well, or if you are coming into work every day worried about your family members being unwell, you can’t be well for everybody that we serve or for the employees that surround you*”. (CS and E, 118).

All three types of participants reported how taking time away from work can foster self-care and allow survivors to maximize their health and well-being, which was important for recovery. One cancer survivor voiced the common sentiment, “*now it’s time to focus on my health and not worry about it (work)*”. (CS, 107). “*And I honestly, I don’t know how anybody can go through treatment and work full-time and do a good job at both*”. (CS, 104) A healthcare provider stated, “*And my threshold is pretty low for telling patients they don’t have to work because it’s a lot, going through radiation, from a physical standpoint, from a mental standpoint, from a financial standpoint, emotional standpoint… you’re dealing with your own mortality. And sometimes I think it’s important to just take time off and take time for yourself and I’ll write a letter to their employer more or less stating those things exactly. And that usually is enough to get them out of work for a few weeks*”. (HP, 203).

It is important to note that of the three types of participants, cancer survivors were the only ones who also focused on the benefits of working, such as distraction from treatment or experiencing a feeling of mastery or control in the worker role. That said, there could be ambivalence about working, as expressed by this survivor: “*…on the one hand I was grateful for the flexibility (in work schedule). On the other hand, I was sort of resentful that I even had to work while I was going through this. And on the third hand, um, I think it really helped me to work because I, you know, I love what I do and you know, for me, I was thinking about it as like I’m really fighting for my life and so what is my life? My life is family and my loves and my work. And so I may as well really live the life I’m fighting to live while I’m fighting to live it, you know? (laughs). So, um, so it was—it was also okay that—that I worked. And helpful. I could see myself really spiralling into depression if I hadn’t been working, like just sitting—sitting home and like sort of wallowing in like, ‘oh my God, I have cancer. Oh my God, I’m—I’m doing chemo’. And work kept me focused on some of the things I loved doing*”. (CS, 112).

For those still working, the ability to reduce or flex their schedule was important. “*Um, this, you know, they were wonderful about scheduling [treatment] around my teaching schedule. Being able to book those four o’clock appointments, you know, for those 20 (chemotherapy) rounds was, was great*”. (CS, 113). An employer stated, “*I think just like the best thing is the flexible time, because it allows them, if they wanted to leave work and rest for like a couple hours and then return to work. I think that is a huge resource to have. I mean, other than that, there’s not much resources that we can provide*”. (E, 308).

While time off was mentioned as being helpful, those who are self-employed may not have the luxury of paid time off. One cancer survivor discussed that she had more support when her cancer recurred and she was employed by a company than she did during her initial diagnosis that occurred when she owned her own business. “*Definitely having the security of…maybe take one day off and just curl up in my bed because I had what they called sick time, where I didn’t have that before (when self-employed as a business owner)…Um, so it was difficult, very scary, very financially troubling, uh, because there is no safety net for self-employment*”. (CS, 109).

### 3.4. Theme 3: Lack of Information

The third theme that ran through the interviews was that each type of participant discussed a lack of knowledge about some aspect of employment support. For cancer survivors, the knowledge gap could be about terminology. One cancer survivor stated, “*…I don’t identify as having a ‘disability,’ but I fit into the criteria so I got all like tripped up on the vocabulary and no one actually verbally explained it. So I’m getting all this, this form with, in bold, ‘Americans with Disabilities’…but I just shredded it because I’m like, I don’t have a disability…honestly, even though like I would consider myself to be well educated… (I) didn’t know the difference between an accommodation and a restriction*” (CS, 101). For other cancer survivors, it was hard to imagine what resources were available. “*So I think if employers could put together… tips and things like that, and strategies… give examples… when it comes back to something that I can access, I ought to have a toolkit, right? … So, yeah, I think if they gave a toolkit and gave some examples of things like that and being transparent instead of being hidden about it, that would be helpful of, “You’re returning back to work. Here’s some examples of some accommodations”, because it’s all kind of not talked about. So I don’t even know where to start with it. And even when they give me a blank canvas to say, “You go talk to your provider”, I don’t even know what to ask my provider for, like, “What do I need?” I don’t even know what I need…*” (CS, 123). That sentiment that “*…sometimes you don’t even know what you need*” (CS, 117) was voiced by many cancer survivors.

For both cancer survivors and healthcare providers, sometimes they lacked knowledge about the regulations regarding disability and laws supporting employees’ rights. Many mentioned that aspects of employment regulations and supports are complicated and not well-integrated into cancer care. “*I contacted Triage Cancer just a few days ago, and I said, “It’s really ambiguous to me about when we should have people apply for SSDI (Social Security Disability Insurance)…And this is one of the attorneys who I took a day long course from a few years ago. And she said, ‘It’s really, really complicated*”. (HP, 201). Many healthcare providers’ knowledge of what can be done to support employment is limited. “*Uh, I mean, as, as providers, we need more education on what, um, constitutes safe employment for a cancer survivor… Um, there are medical specialties where this is the main focus, on how strong the patient is, how agile or what their balance is. So in, in oncology, we don’t do this in any kind of prescribed fashion. Maybe, maybe that’s what I feel we may need more education, how to assess. And the second thing is, uh, knowledge of resources*”. (HP, 206). Another healthcare provider stated, “*(I) think it would be lovely for patients to know—or have a more global support maybe for like, okay, returning to work. Like, here’s someone to talk with or some resource, like, as you’re thinking about going back to work. Um, you know. A more standardised set of either resources or someone that they could go to specifically about these kinds of questions that come up rather than me who doesn’t really know. You know? And all I can do is say like, yeah, you’re—you’re gonna be really tired and working eight hours on your feet is probably not gonna work for you. But then they’re like I have to go back and I have to pay the bills… It’s what’s challenging. I do feel a little bit inadequate sometimes*”. (HP, 210).

Participants noted that employers and co-workers do not always know much about the lived experience of cancer treatment and survivorship. A cancer survivor discussed how a person from human resources remarked that other employees with cancer had taken less time away from work: “*…the HR director said… well, we don’t wanna set a precedent…we’ve never had somebody out this long for cancer and then… I guess just some, some educational pieces (are needed) on… the fact that, you know, not all cancers are the same. Not all, and then even the same exact cancers and, and different people at different ages, um, have different outcomes. They have different ways that they react to their medicines and different, you know, mentally or in a different place maybe with their life. And it’s just not to, to lump everybody together in this one same thing because that’s not, you know, it’s not that way for any disease… And I think some educational pieces about like the anxiety that an employee feels that next time they get sick and, and worrying that it’s cancer again. Or, the fact that they’ve been out for so long and that they worry about their (job.)*” (CS, 116). An employer echoed that sensitivity to the lived experience of cancer might be important for employers., saying education is needed regarding “*…what it’s like for somebody to deal with a cancer diagnosis… of how unrelenting it is and how, yeah. There’s ups and downs… So I think probably some education about, um, what the path looks like, and that they (cancer survivors) probably need support along the path. So I think that kind of education for employers would be really helpful*”. (E, 302).

Finally, there are resources that are available to cancer survivors, but the participants need to know how to find the resources. “*… I think people can go online and Google things and they can find resources like the Job Accommodation Network and Triage Cancer and other organizations that kind of specialise in supporting patients around employment issues stemming from their cancer diagnosis… They do have to know to go look for something and have the—the technology available to be able to—to search for that, but, um, not everybody does. Not everybody knows that there might even be options, um, so that’s a big problem in my opinion*”. (HP, 211) Beyond that, healthcare providers also need to know there are resources and ways to support survivors. “*I think we don’t have a lot of resource… a lot of those programs are if people are trying to stop working or get a break from work for chemotherapy, it’s less focused on (keeping) their jobs, I think*”. (HP, 214). Finding resources and asking for help can be hard when a survivor is more reticent or overwhelmed by the sheer amount of information that they are processing. “*Some patients really don’t ask a lot of questions and don’t really internalize what we’re telling them and then they’re surprised by their side effects or surprised by how many treatments that they have to go through. And so I think education is a really big part of it*”. (HP, 203).

## 4. Discussion

Three themes echoed throughout these interviews: (1) cancer survivors shoulder the greatest burden in identifying and articulating work-related issues; (2) time away from work and flexibility with scheduling work and treatment activities are the primary accommodations requested by cancer survivors and offered by employers; and (3) there are gaps in knowledge regarding ways to support employment goals of cancer survivors. We heard aspects of each theme when talking to cancer survivors, healthcare providers, and employers.

Regarding the first theme, our participants reported that cancer survivors themselves were primarily responsible for both initiating conversations about employment challenges and telling healthcare providers and employers what type of support they needed regarding those challenges. Our findings echo other studies that highlight the importance of cancer survivors’ consistent and open communication with employers [14,20] and studies in which employers appreciate the privacy needs of employees but explain that it is hard to fully support their employees without a better understanding of the challenges they are facing [17]. This also resonates with other studies that have found that personal traits such as confidence, conscientiousness, the ability to explain limitations about one’s capacity and set boundaries were facilitators of positive work experiences during and after cancer treatment [16]. However, ours is not the first study to find that it can be hard for cancer survivors to predict how they will feel and what they will need from employers [15]. The experiences of our participants suggest that expecting cancer survivors to articulate what they need may place an onerous burden upon them. Our results suggest two practical strategies that could reduce this burden. First, screening for work-related distress during healthcare encounters could reduce the burden on cancer survivors to initiate conversations about work. Second, having access to vocational specialists who could act as advocates for cancer survivors and interface with employers and professionals might serve as additional support for cancer survivors, who, in our study, bore the greatest burden in identifying and resolving work issues.

Regarding the second theme, the focus on providing time off from work as a primary method to support cancer survivors is also reported in international research. Research in France indicated that the most common workplace accommodations for cancer survivors involved the reduction of working hours or modification of working schedules, reported by 49% and 42% of cancer survivors who received accommodations, respectively [21]. Reduced hours and flexible scheduling were also noted as the main type of accommodation in a study from Canada [17]. However, in the Canadian study, they also discussed other accommodations such as modification of duties, job retraining, the introduction of technologies or aids, and environmental modification [17]. This indicates that more options are available to cancer survivors and employers, but there may be less awareness about other accommodations that could be deployed. Identifying other accommodations may be particularly important in light of the ambivalence voiced by many cancer survivors as they both articulated reasons for wanting to take time away from work and reasons for wanting to work during treatment. Other studies of cancer survivors indicate that cancer survivors often have a strong desire to maintain employment [16]. People living with metastatic cancer who want to keep working can also be left out of this conversation if the prevailing mindset is to leave the workforce during cancer treatment and return when finished with treatment. Further, even those who want and need time away from work may struggle when returning to work or increasing their hours. For example, breast cancer survivors in Ireland take an average of 11 months off from work during breast cancer treatment but still report challenges and the need for employment support programs when returning to work [22]. Providing release time from work as the only employment support implies that all cancer-related challenges will resolve with time and self-care, yet this contradicts recent trends to characterise cancer as a chronic illness [23]. Furthermore, time off from work may result in additional social isolation and a lack of behavioural activation, which could promote feelings of depression and social withdrawal. As such, it may behoove us to implement programs that allow cancer survivors and employers, with the support of healthcare providers as needed, to create customised return to work plans that address a full range of workplace accommodations [24].

Regarding the third theme, the knowledge gaps we heard in this study related to many aspects of supporting employment were also seen in other studies. Lack of knowledge about cancer and its treatment and discomfort in talking about it has been reported in another qualitative study of employers and co-workers [12]. Likewise, a consensus-building study of cancer survivors and healthcare providers noted a need for training and education in employment support, as healthcare professionals have limited training in this topic [25]. Cancer survivors likewise have voiced a lack of information regarding how cancer affects employment and what they can do about it [26]. Many participants voiced the need for a toolkit. There are existing resources such as the Cancer and Careers organization (www.cancerandcareers.org (accessed on 19 May 2022)), the Job Accommodations Network (www.askjan.org (accessed on 19 May 2022)), and Triage Cancer (www.triagecancer.org (accessed on 19 May 2022)) which have resources including workbooks and resume services. Those resources should be publicised in many different places where cancer survivors may be likely to encounter them, such as cancer centre waiting rooms, healthcare offices, support groups, and social media. To supplement these existing resources, local information on state-specific disability and employment laws and how they apply to different workplaces would benefit cancer survivors and their employers.

The three themes highlight one of the difficulties of supporting employment: no one person, structure, or discipline “owns” this problem. Each of the stakeholders we interviewed had a perspective on part of the problem, e.g., cancer survivors understood their job and their lived experience, employers understood their needs, the workplace, and the rules that they need to follow, and healthcare providers understood the nature of the disease and the ways in which symptoms and side effects may be ameliorated and when they might not be able to be fully resolved. Additionally, each of them had a different set of priorities and constraints that affected their actions. Future research needs to continue to explore and test ways in which policies, technologies, and programs can foster optimal communication and collaboration between these three types of stakeholders.

Finally, some limitations of the study design should temper the interpretation of these data. Most importantly, because we were focused on people living within the catchment area of our academic medical center, the sampling strategy did not result in a large, diverse sample reflective of the national population of cancer survivors. Additionally, we found it challenging to recruit an adequate sample of employers, as many told us they were having a hard time recollecting any instances where their employees were undergoing treatment. As such, it is best to view the results from this convenience sample as reflective of experiences in the local context of one cancer centre in northern New England.

## 5. Conclusions

These results suggest that cancer survivors have resources available to them but need to exert energy and initiative to identify and mobilize those resources. In doing so, they may need to solicit and synthesise information from multiple places and people, all of whom have a somewhat narrow view of work challenges and the options to minimise those challenges. Facilitating access to curated resources followed by individualised problem-solving and application may be one option for reducing the burden shouldered by cancer survivors regarding employment optimization.

## Figures and Tables

**Table 1:** Participant characteristics (*N* = 45).

Characteristic	Cancer Survivors	Healthcare Providers	Employers
**Gender**			
Female	19	14	3
Male	4	3	2
**Age**			
Average	55.0	51.7	51.4
Range	28–78	27–74	34–61
Std. Deviation	11.0	12.1	10.7
**Ethnicity**			
Hispanic or Latino	0	0	0
Non-Hispanic or Latino	23	17	5
**Race**			
American Indian/Alaska Native	0	0	0
Asian	0	1	0
African American/Black	0	0	0
Native Hawaiian/Pacific Islander	0	0	0
White	23	16	5
**Education**			
<12 years (did not graduate)	0	0	0
12 years or completed high school	1	0	1
Post high school training other than college (vocational or technical)	1	0	0
Some college	4	0	2
College graduate	7	3	0
Postgraduate	10	14	2
**Current Employment Status ^a^**			
Full-time	14	12	7
Part-time	6	5	1
Retired	0	0	0
Homemaker	0	0	0
On long- or short-term disability	2	0	0
Unemployed (looking for work)	0	0	0
Unemployed (not looking for work)	0	0	0
Other	1	0	0
**Cancer Staging**			
0	0	N/A	N/A
i	4	N/A	N/A
ii	6	N/A	N/A
iii	5	N/A	N/A
iv	5	N/A	N/A
Unsure	3	N/A	N/A
**Treatment Underwent**			
Surgery only	1	N/A	N/A
Chemotherapy only	2	N/A	N/A
Radiation only	0	N/A	N/A
Surgery & chemotherapy	5	N/A	N/A
Surgery & radiation	2	N/A	N/A
Surgery, chemotherapy, & radiation	13	N/A	N/A
**Completed Treatment**			
Yes	17	N/A	N/A
No	6	N/A	N/A
**Occupation**			
Physician	N/A	5	N/A
APRN	N/A	4	N/A
RN	N/A	2	N/A
Social Work	N/A	3	N/A
OT	N/A	1	N/A
Psychologist	N/A	1	N/A
Dietician	N/A	1	N/A
**Job Classification ^a^**	N/A	N/A	
Manufacturing	N/A	N/A	1
Finance	N/A	N/A	1
Non-profit	N/A	N/A	1
Government-run (state or local)	N/A	N/A	2
Healthcare	N/A	N/A	1
Biotech or medical research	N/A	N/A	1
Other	N/A	N/A	1
**Role ^a^**	N/A	N/A	
Owner	N/A	N/A	2
Supervisor/manager	N/A	N/A	3
Human resources	N/A	N/A	3
Occupational medicine	N/A	N/A	0
Other	N/A	N/A	0
**Organization Size ^a^**	N/A	N/A	
Micro (1–9 employees)	N/A	N/A	1
Small (10–49 employees)	N/A	N/A	1
Medium (50–249 employees)	N/A	N/A	2
Large (250+ employees)	N/A	N/A	4
**Organization Ownership ^a^**	N/A	N/A	
Locally owned	N/A	N/A	4
Regional	N/A	N/A	1
Part of a national company	N/A	N/A	1
International	N/A	N/A	2

^a^ These categories include the three cancer survivors who offered their perspectives as an employer in addition to a survivor; this is why these add to *n* = 8.

## Data Availability

The de-identified dataset used and/or analysed during the current study is available from the corresponding author upon reasonable request.
